# Pathogenesis and diagnosis of delayed-type drug hypersensitivity reactions, from bedside to bench and back

**DOI:** 10.1186/s13601-015-0073-8

**Published:** 2015-09-03

**Authors:** Rik Schrijvers, Liesbeth Gilissen, Anca Mirela Chiriac, Pascal Demoly

**Affiliations:** Laboratory of Clinical Immunology, KU Leuven, Leuven, Belgium; Department of Internal Medicine, University Hospitals Leuven, Leuven, Belgium; Department of Dermatology, University Hospitals Leuven, Leuven, Belgium; Exploration des Allergies, Département de Pneumologie et Addictologie, Hôpital Arnaud de Villeneuve, University Hospital of Montpellier, Paris, France; Sorbonne Universités, UPMC Paris 06, UMR-S 1136, IPLESP, Equipe EPAR, 75013 Paris, France

**Keywords:** Drug allergy, Drug hypersensitivity, pi-concept, Hapten, Altered self-repertoire model, Lymphocyte proliferation assay, Pharmacogenomics, Abacavir hypersensitivity syndrome, Tissue-resident memory T-cells, Regulatory T-cells

## Abstract

Drug hypersensitivity reactions (DHR) have been present since the advent of drugs. In particular T-cell mediated delayed-type hypersensitivity reactions represent a heterogeneous clinical entity with a diverse pathogenesis and result in a considerable burden of morbidity and mortality not only driven by the reactions themselves but also by the use of alternatives which are sometimes less effective or even more dangerous. Diagnostic procedures rely on clinical history, skin testing and potential provocation testing, whereas validated in vitro diagnostic procedures are still lacking for most of them. Recent work in the field of pharmacogenomics combined with basic scientific research has provided insights in the pathogenesis of abacavir and carbamazepine hypersensitivities linked with certain human leucocyte antigen risk alleles. Nevertheless, important scientific questions on how other DHR arise and how host-drug interactions occur, remain unanswered. Recent work indicates an intricate relation between host, drug and pathogens in severe cutaneous and systemic reactions and provides more insights in the role of regulatory T-cells and viral reactivation in these reactions. In this review we focus on type IV delayed-type DHR, and address recent advances in the pathogenesis, pharmacogenomics, and diagnosis of these reactions with an emphasis on the understandings arising from basic research.

## Background

Drug hypersensitivity reactions (DHR) are defined as adverse events resembling clinical allergy to an otherwise safe and effective therapeutic agent. Only those in which an immunological mechanism can be demonstrated, mostly after an allergy workup, are termed drug allergies [1]. They constitute approximately 15% of all adverse drug reactions and affect more than 7% of the general population [2]. They result in a considerable morbidity and mortality and will remain to be so in everyday patient care with the ever-increasing armamentarium of drugs. DHR have been present since the advent of drugs. Still, validated diagnostic procedures are lacking for many of them. Moreover, important scientific questions on how these DHR arise and how host-drug interactions occur, remain to be elucidated.

Clinically, DHR are classified as immediate (typically <1 h following the last intake of the culprit drug) or delayed-type DHR (DTH, typically >1 h to days after the start of a treatment with the culprit drug) [2]. Immediate reactions present as urticaria, angioedema, bronchospasm or anaphylaxis, whereas for DTH the clinical spectrum is much wider ranging from fixed drug eruption (FDE), maculopapular eruption (MPE), general exfoliative dermatitis or erythroderma, drug reaction with eosinophilia and systemic symptoms (DRESS syndrome), drug-induced hypersensitivity syndrome (DIHS), acute generalized exanthematous pustulosis (AGEP), Stevens-Johnson syndrome (SJS), toxic epidermal necrolysis (TEN), other bullous reactions mimicking pemphigus vulgaris or bullous pemphigoid up to vasculitis (Table 1). In particular erythroderma, DRESS, AGEP, SJS and TEN, often referred to as severe cutaneous adverse reactions (SCARs), are potentially life-threatening, with an estimated mortality rate of 5–15, 10, 5, 1–5 and 20–30% respectively [3]. Internal organs can be affected as well, either alone or with cutaneous symptoms (as in DRESS and SJS/TEN), and may include hepatitis, nephritis, pneumonitis and cytopenias.

**Table 1:** Classification of DHR according to Gell and Coombs and adapted by Pichler et al. [4]

Type	Type of immune response	Pathophysiology	Clinical symptoms	Typical chronology of the reaction
I	IgE	Mast cell and basophil degranulation	Anaphylactic shock, Angio-oedema, Urticaria, Bronchospasm	Within 1–6 h after the last intake of the drug
II	IgG and complement	IgG and complement-dependent cytotoxicity	Cytopenia	5–15 days after the start of the eliciting drug
III	IgM or IgG and complement or FcR	Deposition of immune complexes	Serum sickness, urticaria, vasculitis	7–8 days for serum sickness/urticaria 7–21 days after the start of the eliciting drug for vasculitis
IVa	Th1 (IFNγ)	Monocytic inflammation	Eczema	1–21 days after the start of the eliciting drug
IVb	Th2 (IL-4 and IL-5)	Eosinophilic inflammation	MPE, DRESS	1 to several days after the start of the eliciting drug for MPE 2–6 weeks after the start of the eliciting drug for DRESS
IVc	Cytotoxic T-cells (perforin, granzyme B, FasL)	Keratinocyte death mediated by CD4 or CD8	FDE, MPE, SJS/TEN, Pustular exanthema	1–2 days after the start of the eliciting drug for fixed drug eruption 4–28 days after the start of the eliciting drug for SJS/TEN
IVd	T-cells (IL-8/CXCL8)	Neutrophilic inflammation	AGEP	Typically 1–2 days after the start of the eliciting drug (but could be longer)

Table adapted from [2].

DHR are classified according to the Gell and Coombs classification, with type IV hypersensitivity reactions accounting for the T-cell mediated, and the majority of DTH. Pichler et al. has subdivided type IV reactions into 4 groups according to the clinical presentation and the involvement of different types of drug-responsive T-cells (Table 1) [4].

In this review we focus on type IV DTH and address recent advances in the pathogenesis, pharmacogenomics, and diagnosis of these reactions with an emphasis on the understandings arising from basic research.

## Pathogenesis

**Fig. 1:**
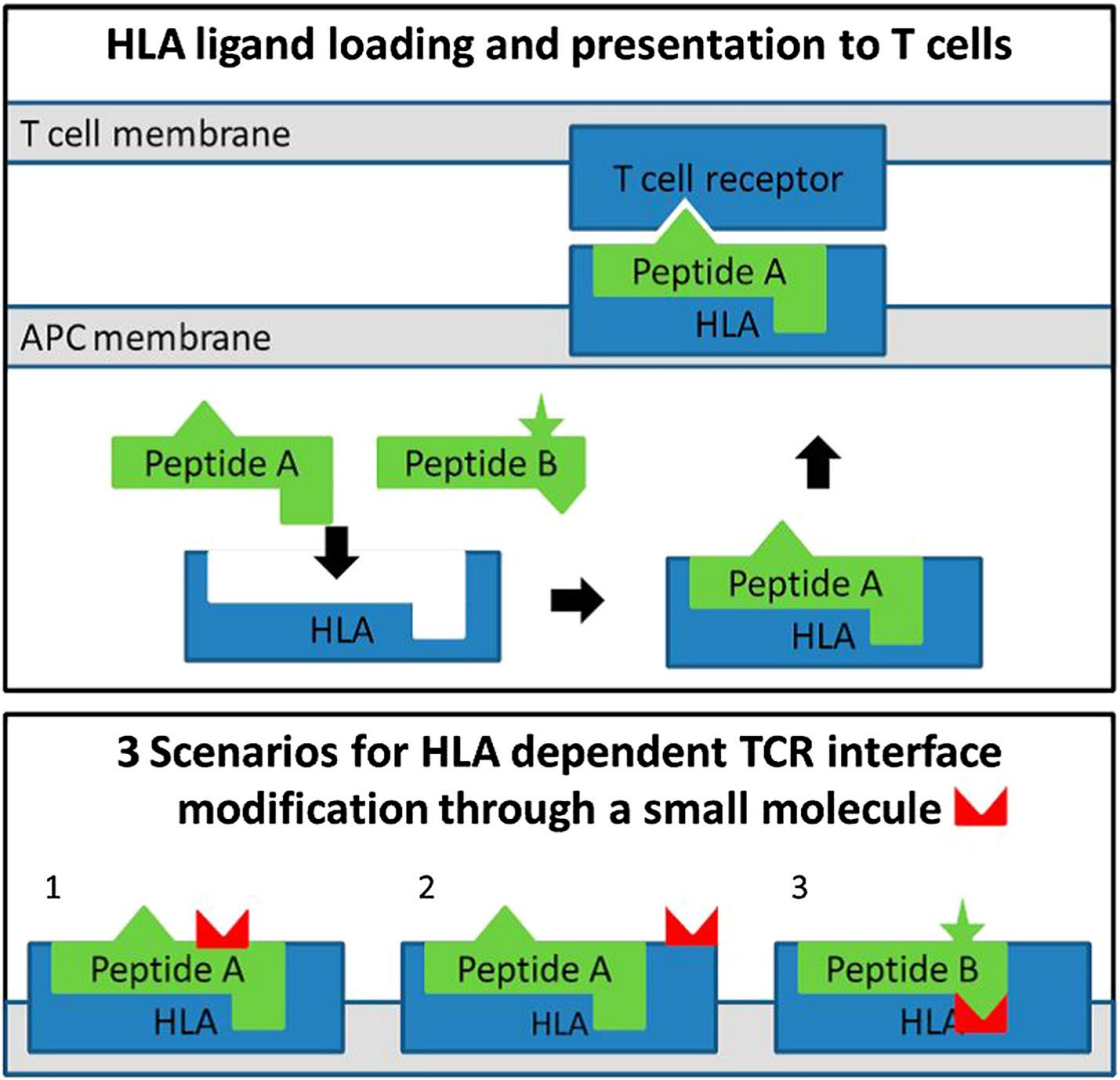
Schematic examples for the (pro)hapten hypothesis, p-i concept and altered self-repertoire model (adopted from Ostrov et al. [16]). *Upper part* TCR monitor the antigens or ligands presented by the HLA molecules. These HLA ligands are typically peptides loaded onto the HLA molecule inside the antigen-presenting cells and subsequently exposed on the surface. Different allelic variants of HLA molecules result in different binding specificities and a specific profile of presented ligands. Here, peptide A, but not peptide B, can bind to the HLA molecule. Typically, T-cells do not react to presented self-peptides, as these auto-reactive T-cells are negatively selected during thymic development, but will react once they encounter an unknown ligand (e.g., a virus-derived peptide). In the *lower part*, three scenarios in which drugs can result in a HLA-dependent reaction: in (*1*) a HLA-specific drug haptenated peptide is presented, according to the *(pro)hapten hypothesis*; in (*2*) the HLA molecule itself is modified in a region exposed to the TCR, resulting in a reaction according to the *p*-*i concept*; and in (*3*) the binding specificity of the HLA molecule is altered by the presence of the drug, resulting in presentation of novel ligands such as peptide B, as in the *altered self*-*repertoire hypothesis.*

Scrutinizing the pathogenesis of DTH focuses on the interaction of drugs with the immune system. Multiple mutually non-exclusive hypotheses explaining the pathogenesis of DHR, including DTH, exist and include the (pro)hapten hypothesis, the pharmacological interaction (p-i) concept and the altered self-repertoire model (summarized in Fig. 1), as well as the danger model. For most models, examples have been elaborated and some proofs exist.

First, a drug can act as a hapten (or prohapten, if a reactive metabolite acts as the hapten) whereby covalent binding to proteins or other larger molecules results in the formation of an antigen. This *(pro)hapten hypothesis* is still considered the primary pathway by which chemical sensitizers cause allergic contact dermatitis, and it is also identified as a well-known pathway for β-lactam hypersensitivity [1, 5]. Using mass spectrometry, conjugates of β-lactam antibiotics, such as benzylpenicillin derivates, but also piperacillin, meropenem, or aztreonam, bound to specific lysine residues on serum albumin resulting in the formation of an antigenic epitope, were identified and/or detected in patient plasma [6]. Next, synthetic β-lactam-albumin conjugates were able to stimulate previously identified drug-specific T-cell clones in an in vitro lymphocyte proliferation assay (LPA) [7, 8], corroborating with the (pro)hapten hypothesis in DTH [9].

Second, according to the *p*-*iconcept* [the pharmacological interaction with immune receptor (p-i)], certain drugs interfere with the antigen presenting process, without the requirement of a specific peptide ligand, but through non-covalent interaction with human leucocyte antigen (HLA) alleles and/or T-cell receptors (TCR), to trigger an immune response [10]. This hypothesis enables to explain why some drugs, for instance contrast media, would generate an eruption already at the first contact, i.e. without a known prior sensitization step. Here, pre-activated drug-specific T-cells present in these patients are hypothesized to display cross-reactivity for the drug-antigen presenting complex and circumvent the classical antigen processing steps [11]. Yang et al. failed to demonstrate carbamazepine-modified peptides presented by HLA-B*15:02, a known risk allele for carbamazepine-induced SJS/TEN in certain Asian populations [12], but detected HLA-B*15:02-specific peptides and carbamazepine alone, suggesting a non-covalent interaction [13]. Next, allopurinol- or its metabolite oxypurinol-specific T-cell lines were demonstrated to react immediately (after seconds to minutes) to the addition of the drug, bypassing intracellular antigen processing—as demonstrated by the lack of inhibition by pretreatment with a proteasome inhibitor—and were not limited to a particular TCR Vβ-pattern, consistent with the p-i concept [14]. An increased affinity of oxypurinol to the peptide-binding groove of HLA-B*58:01 was calculated in silico, and put forward as an explanation for the increased risk for allopurinol-induced DTH associated with this specific HLA-type [14].

**Fig. 2:**
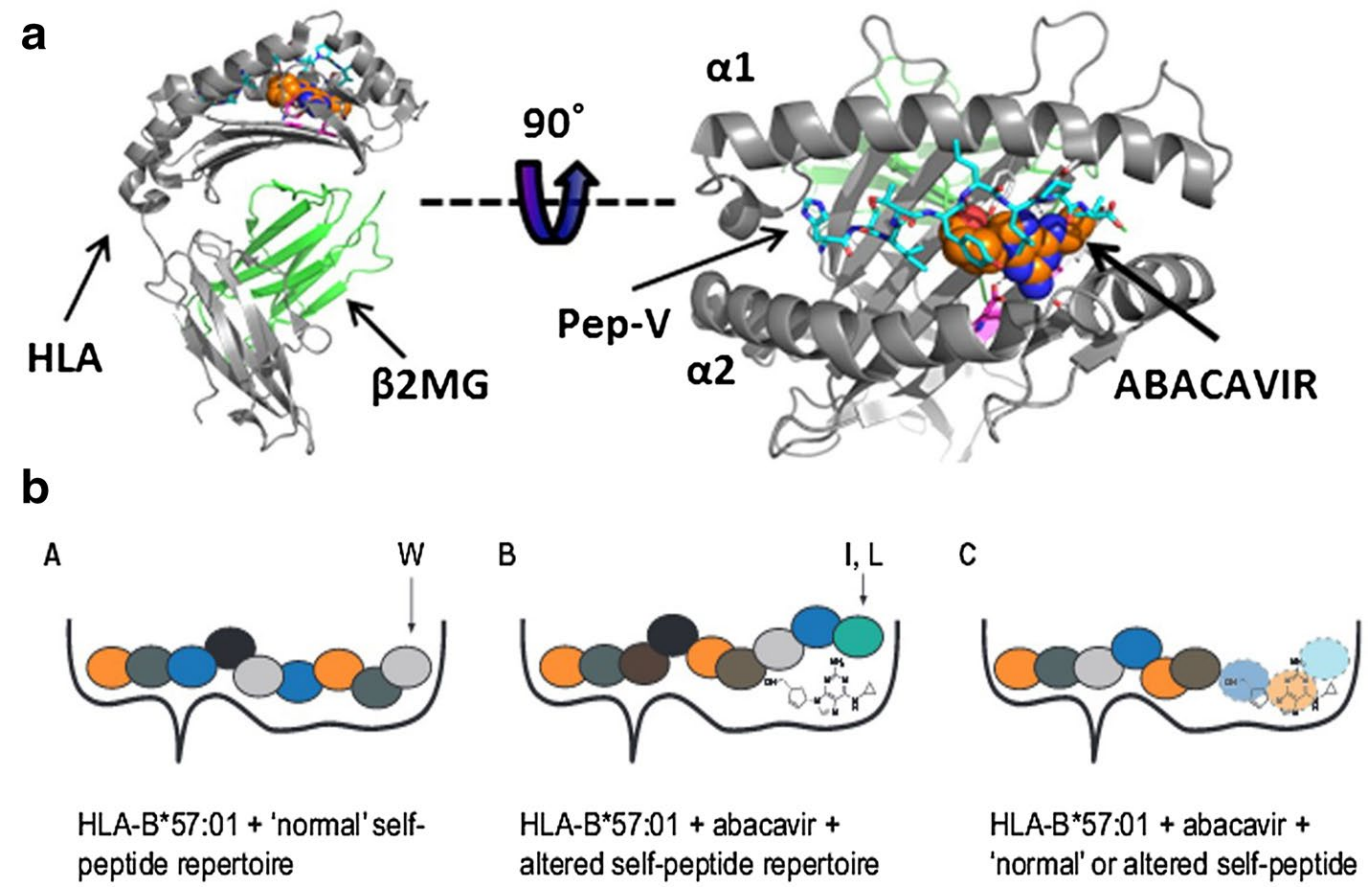
Abacavir-induced altered self-peptide presentation (adopted from Ostrov et al. [16] and Yun et al. [25]). **a** Crystal structure of a peptide, HSITYLLPV or Pep-V (*cyan*) bound to HLA-B*57:01 (*gray*) together with abacavir (shown as *spheres*, *orange* for carbon, *blue* for nitrogen, and *red* for oxygen) [16]. **b** Without abacavir, the HLA-B*57:01 presents the ‘normal’ self-repertoire peptides and thus does not trigger an immune response (*left*). In the presence of abacavir, the drug can be incorporated in the F pocket of the HLA molecule thereby altering the peptide repertoire that is loaded onto this molecule. Abacavir binding favors binding of peptides with tryptophan (W) or phenylalanine at the C-terminus (position 9) rather than the small aliphatic residues (e.g., valinine, alanine, or isoleucine) which are normally bound to unmodified HLA-B*57:01 [16, 28]. This results in recognition of neo-antigens by neo-antigen primed CD8+ T-cells (*middle*). On the *right*, a hypothetical model of how abacavir might result in the selection of shorter peptide or is recognized itself by the TCR [25].

Ensuing on the p-i concept, the *altered self*-*repertoire model* emerged based on findings explaining the increased risk for DTH upon abacavir-exposure in HLA-B*57:01 positive individuals. Here, mass spectrometry on HLA-associated peptides in the presence or absence of abacavir along with crystal structure data, demonstrate that abacavir lies across the bottom of the HLA-B*57:01-binding groove, interacting with several peptide binding pockets (C, D, E) but predominantly protrudes into the F pocket, thereby altering the repertoire of usual presented peptides (Fig. 2) [15–17]. Moreover, abacavir interacts with the two residues (D114 and S116) distinguishing HLA-B*57:01 from HLA-B*57:03, thereby explaining the lack of association with the latter HLA-type [16]. The resulting T-cell response is not mono- or oligoclonal as observed in cases where a drug induces a single novel antigenic epitope, but polyclonal as illustrated by the unbiased TCR Vβ-pattern and antigen-binding complementarity-determining region 3 in patient-derived abacavir-selective T-cells [15]. Together these data indicate that the normally self-tolerant T-cell compartment is exposed and activated by neo-self peptides presented by the HLA-B*57:01-abacavir complex. In line, Lucas et al. recently isolated abacavir-specific CD8+ T-cells from abacavir-unexposed healthy HLA-B*57:01 positive individuals from both the memory and naïve CD8+ T-cell compartment, indicating the existence of pre- and/or de novo primed CD8+ T-cells recognizing the abacavir-altered self-peptide via cross-recognition with a hitherto unknown foreign antigen in the case of pre-existing memory CD8+ T-cells [18]. These findings correlate with clinical data demonstrating that HLA-B*57:01 individuals exposed to abacavir can develop a DTH ranging from as early as 48 h up to 6 weeks after the first exposure. However, why only 55% of HLA*B-57:01-positive individuals experience abacavir hypersensitivity, although drug-specific T-cells can be identified in vitro in 100% of HLA-B*57:01 positive and 0% in HLA-B*57:01-negative individuals, remains to be elucidated [18]. A second example for this model comes from studies of the association of HLA-B*15:02 with carbamazepine-induced SJS/TEN. Here, in silico work suggested that carbamazepine likewise binds to the HLA-B*15:02 molecule, yet at secondary anchor sites (i.e., underneath the P4/P6 residues of the presented peptide), resulting in a smaller shift in presented peptides compared with that observed with abacavir in HLA*B-57:01 [15]. Recently, also for dapsone-induced hypersensitivity syndrome, HLA-B*13:01 was identified as a risk-allele [19]. What distinguishes HLA-B*13:01 from other HLA-B*13 alleles are three amino-acid residues (at position 94, 95, 145) located at the peptide-binding groove and binding pocket F, suggesting similarities with the examples of abacavir and carbamazepine. However, further insights in the immunopathogenesis of the dapsone hypersensitivity syndrome remain to be elaborated.

Finally, according to the *danger model *the barrier for the development of DHR may be overcome if other risk factors are present. According to this model proposed by Matzinger [20], the immune system is more concerned with potential danger than foreignness [21]. Therefore an exogenous pathogen or chemical, or an endogenous intracellular molecule released from necrotic cells, might not evoke an immune response unless the immune system detects ‘danger’ [22, 23]. In the absence of danger, tolerance will result. Therefore, it is hypothesized that concomitant exposure to other signals such as chemicals, drugs or infectious agents, can trigger or amplify the innate immune response, resulting in amplification of insufficient stimuli over a critical threshold, thereby enabling DTH to occur [24]. For instance in the case of an infection, inflammatory cytokines that are produced by the innate immune system in response to the pathogen might provide a danger signal that directly or indirectly enables the generation or activation of drug-specific T-cells. On the other hand, this could also explain why in the absence of these co-stimuli, testing for DTH later could remain negative. Although this hypothesis, considered additive to the other models, might be consistent with certain clinical observations, it currently lacks clear experimental evidence. Next, many questions remain unanswered such as why some drugs induce stress and/or cell death, but apparently do not result in an immune reaction as in the example of paracetamol-induced hepatotoxicity [23].

## Pharmacogenomics

During the last decade many associations between HLA molecules and the development of certain DHR have been reported (Table 2 and reviewed in [25]). The strongest associations have been described for HLA-B*57:01 with the abacavir hypersensitivity syndrome, HLA-B*15:02 with carbamazepine-induced SJS/TEN, and HLA-B*58:01 with allopurinol-induced severe cutaneous adverse reactions [26]. Most associations are with class I but also class II molecules are described, both with varying degrees of association [26]. Next, also ethnicity plays a role, possibly reflecting in part the differences in HLA-backgrounds. However, HLA-associations do not explain all cases, indicating additional factors contribute to the mechanisms of drug hypersensitivity. Linkage disequilibrium and the presence of certain TCR clonotypes have been considered as a potential explanation for these discrepancies.

**Table 2:** Well-defined HLA associations in DHR [28]

Drug	Syndrome	HLA allele	HLA carrier rate	NPV	PPV (%)	NNT
Abacavir	ABC HS	B*57:01 [31, 34]	5–8% Caucasian	100%	55	13
Allopurinol	SJS/TEN and DRESS/DIHS	B*58:01 [25]	9–11% Han Chinese 1–6% Caucasian	100% in Han Chinese	3	250
Carbamazepine	SJS/TEN	B*15:02 [12, 27, 28]	10–15% Han Chinese	100% in Han Chinese	3	1,000
Dapsone	DRESS/DIHS	B*13:01 [25]	28% Papuans, Australian aborigines; 2–20% Chinese; 1.5% Japanese; 1–12% Indian; 2–4% Southeast Asians	99.8%	7.8	84

*NPV* negative predictive value, *PPV* positive predictive value, *NNT* numbers needed to test (to prevent one case), *ABC HS* abacavir hypersensitivity syndrome.

Carbamazepine hypersensitivity is found to be associated with HLA-B*15:02 in most Asian populations, but not Japanese or Korean, and with HLA-B*31:01 in Europeans, although this was not reported consistently in all studies [12, 27, 28]. In the Han Chinese population, HLA-B*15:02 was present in 100% of the SJS/TEN patients [29]. However this risk-allele was also found in 3% of carbamazepine-tolerant individuals, in 8.6% of the general population, and no association was observed in Caucasian or Japanese populations, although here a lower incidence of HLA-B*15:02 is observed, nor was there an association with carbamazepine-induced MPE, indicating additional associated risk factor(s) are yet to be identified [12]. Ko et al. provided strong evidence that in addition to the associated HLA allele, particular TCRs play a role in the development of the immune response in the case of carbamazepine-induced SJS/TEN in HLA-B*15:02 positive individuals [30]. They identified a skewed and restricted TCR usage in affected patients and identified these clonotypes in blister fluid cells from patients. The identified TCR clonotypes (predominantly Vβ-11-ISGSY and Vβ-11-GLAGVDNY) were absent in 11 carbamazepine-tolerant subjects of whom 2 carried the HLA-B*15:02 risk allele. Moreover, the two clonotypes could be observed in respectively 4/29 (14%) and 2/29 (7%) of healthy carbamazepine-unexposed HLA-B*15:02-positive subjects in whom a cytotoxic response could be identified in vitro in the presence of carbamazepine, suggesting these patients would develop a SJS/TEN if exposed in vivo. The in vitro cytotoxic response could be blocked with an antibody against TCR-Vβ-11, suggesting routes for future therapeutic strategies. Of note, these findings are consistent with the observations of a smaller shift in presented peptides by HLA-B*15:02 upon exposure to carbamazepine compared with the shift observed with HLA-B*57:01 exposed to abacavir in vitro [15]. In the former, a smaller shift of presented peptides might also explain a more biased T-cell response. However, also direct interaction, without the presence of loaded altered peptides, has been suggested for carbamazepine and HLA*B-15:02 [28].

Abacavir hypersensitivity is associated with HLA-B*57:01 probably in most ethnic groups [31]. However, it is a rare HLA-type (<1% [32]) in Taiwanese or Korean populations where abacavir hypersensitivity is less frequent and might be mediated via other mechanisms [32, 33]. DHR after abacavir exposure are potentially life-threatening, CD8+ T-cell mediated, HLA-B*57:01-restricted, and previously occurring in ~5% of treated individuals [34]. HLA-B*57:01 screening has a negative predictive value (NPV) of 100% for abacavir hypersensitivity syndrome and a positive predictive value (PPV) of 58% [34, 35], precluding further use of abacavir in case of positivity.

Next, HLA-B*58:01 is associated with a risk for allopurinol (or its metabolite oxypurinol) induced DRESS or SJS/TEN, mostly in Han Chinese [14] with a PPV of 2.7–18% [25].

Recently, HLA-B*13:01, present in 28% of Papuans and Australian aborigines, 2–20% of Chinese, 1.5% of Japanese, 1–12% of Indian, 2–4% Southeast Asians but largely absent in Europeans and Africans, was identified as a risk factor for dapsone hypersensitivity syndrome, developing in 0.5–3.6% of treated individuals. HLA-B*13:01 had an estimated PPV of 7.8% and NPV of 99.8% [19].

The findings from pharmacogenomic studies are highly translational. Current HIV guidelines recommend HLA-B*57:01 testing prior to initiating abacavir treatment [36]. The Food and Drug Administration recommends screening for HLA-B*15:02 before starting a treatment with carbamazepine in patients with ancestry in at-risk populations [37]. Also, the use of a HLA library containing different HLA molecules to screen new drugs for their ability to bind these molecules as a screen for their potential to cause severe DHR has been proposed [38]. The development of congeners that retain pharmacological activity, but do not cause immune reactions could be imagined [2].

## Relation between host, drug and pathogen

Recent work suggests an intricate relation between the host, drug and pathogen in severe cutaneous and systemic reactions, in particular in DRESS or DIHS [26]. Drugs typically involved in DRESS comprise antibiotic sulphonamides, anticonvulsants, β-lactam antibiotics, allopurinol, NSAIDs, and nevirapine and for most of these drugs, an association with the reactivation of latent human herpes viruses (HHVs such as EBV, CMV, HHV-6, and HHV-7), in some cases sequentially [39], has been observed [40, 41]. However, not always cell free viral load but also increase in virus-specific immunoglobulins have been used as a measure for viral reactivation [42–44]. Whether HHV reactivation is a complication or an innocent bystander phenomenon rather than a cause of drug-induced DRESS/DIHS remains a matter of debate [26, 43]. HHV reactivation may be associated with more severe reactions [45] and viral reactivation correlated with the degree of inflammation [41]. Reactivation can be asymptomatic, but is also associated with prolonged symptoms in DRESS, long after stopping the causative drugs, or may cause organ-specific viral disease [46]. The demonstration of HHV reactivation may be a useful marker for the diagnosis of DRESS, and has been added to the DRESS-scoring criteria in Japan [39]. In contrast to DRESS/DIHS, viral reactivation seems to be uncommon to occur in other severe cutaneous DHR such as SJS/TEN, AGEP.

What might be the relation of viral reactivation with DRESS/DIHS? It has been hypothesized that in the presence of viral replication, co-stimulatory molecules are up-regulated, lowering the threshold that is required for T-cell activation. Also, virus-specific T-cells from previous immunization may cross-react with drug-altered HLA-presented peptides according to the *heterologous immunitymodel* [28]. Picard et al. demonstrated expanded populations of CD8+ T-cells from DRESS patients recognizing EBV epitopes [47], although this awaits further independent confirmation [26]. Next, the drug may interact more favorably with viral peptides loaded onto HLA molecules, stimulating T-cell responses [25]. Also, the direct activation of virus production by culprit drugs has been suggested and demonstrated for EBV in EBV-transformed B-lymphocytes from patients with DRESS [47]. Finally, when the causative drug or concomitant drugs induce a degree of immunosuppression, the altered immune conditions might facilitate reactivation of latent HHVs.

## Skin-resident memory T-cells and regulatory T-cells in DTH

Recent studies have shown that after viral infection, a small fraction of memory T-cells persist in peripheral tissues such as the skin and are considered tissue-resident memory T-cells (Trm, characterized as CD69+ CD103+ CD8+ T-cells) [41, 48, 49]. These skin-resident memory T-cells may also play an important role in DHR, in particular in FDE [41, 50]. For instance, Trm in resting lesions evolved to protect epidermal tissue from invading pathogens might cross-recognize a drug antigen, resulting in localized epidermal damage upon exposure to the eliciting drug. This hypothesis would explain the intriguing observation of why FDE lesions often appear at exactly the same site as a previous HSV infection or trauma [50]. It has also been proposed that Trm cells develop after an abacavir hypersensitivity syndrome as suggested by positive patch testing in 79% of cases versus negative patch testing in HLA-B*57:01-positive yet abacavir-naïve individuals, although in both groups circulating abacavir-reactive T-cells could be identified. The authors suggest a prior systemic reaction to be necessary to generate Trm cells that hence enable positive patch testing [51]. These abacavir-specific-Trm cells however remain to be identified. These findings also indicated that patch testing in HLA-B*57:01-positive abacavir-naïve individuals probably cannot distinguish between those who will and those who will not develop an abacavir hypersensitivity syndrome upon exposure.

FoxP3+ regulatory T-cells (Tregs) suppress effector T-cells and play a pivotal role in the balance between increased susceptibility for infections and autoimmunity and the role of Tregs in DTH is increasingly being recognized. Tregs expand at the acute stages of DRESS/DIHS but decrease and become functionally deficient upon resolution of DRESS/DIHS, possibly explaining the delayed onset and viral activation at the acute stage and the risk of subsequent autoimmune disease upon resolution [52]. Alternatively in SJS/TEN, the suppressive function of Tregs was reduced in the acute stage, possibly reflecting increased epidermal toxicity at this stage [52]. Recently, also a case where drug-specific Tregs were increased during the recovery stage was described [53]. Next, Tregs were demonstrated to play a role in FDE. Tregs migrate to the extending edges of the inflammatory sites of FDE, where they inhibit intradermal CD8+ cytotoxic T-cells and prevent further disease progression [54].

## Diagnosis of DTH

Diagnosis of DTH after systemic administration remains difficult in everyday clinical practice. Currently, once a patient presents with a potential DTH, assessment of causality is based on a clinical judgment evaluating the relationship between drug intake and the time of onset, type of drug and aspect of the adverse reaction. This approach often leads to the eviction of numerous drugs and drug classes and/or the switch towards suboptimal alternatives. Clarification of the culprit drug(s) and/or chemicals, identification of potential cross-reactive molecules and safe alternatives require a complete allergy workup including skin testing [55–57] followed by a drug provocation test if indicated and if no contra-indications exist [2]. These in vivo tests can only be performed after a certain delay (at least after healing of the initial reaction) and are accompanied with the risk for re-eliciting symptoms (locally and/or systemically), fueling research for validated in vitro tests. Moreover, both clinicians and researchers working in the field of DHR agree that there is a need for diagnostic tools, in particular for the diagnosis of severe cutaneous DHR, or those DHR affecting internal organs including the liver, lungs, kidneys, and bone marrow. The development of tools for biological diagnosis is indeed crucial for those cases where a drug provocation test is not possible.

The presence of drug-specific T-cells that are not detected in tolerant (exposed) controls plays a central role in the pathogenesis of almost all known forms of DTH. Therefore, the most commonly used in vitro test, the LPA, aims to detect these drug-specific T-cells as a marker of sensitization. The assay is based on the incubation of freshly isolated peripheral blood mononuclear cells (PBMCs) from hypersensitive patients with titrated concentrations of the suspected drug or a vehicle control enabling the determination of a stimulation/proliferation-index 5–7 days later. Although safe and able to test many different drugs in various types of DTH, the assay is technically demanding, subject to toxicity issues, and unable to evaluate drug metabolites or most drug antigens acting as haptens. Moreover, although specificity of the LPA is high (at least 85% [58]), the sensitivity of the assay is rather low (~60 to 70% [59]) and probably reflects the low frequency of drug-specific T-cells in hypersensitive patients (estimated to be 1:250 to 1:10,000 [60]) and/or insufficiently sensitive read-out. Currently, a DHR cannot be ruled out in case a negative result is obtained. The sensitivity also depends on the type of reaction, with low sensitivities being reported in CD8^+^ T-cell mediated reactions [1].

To improve the sensitivity of this in vitro assay, several strategies have been evaluated recently. Addition of anti-CTLA4 and anti-PDL1 to LPA cultures increased the sensitivity of the assay with more positive LPA assays and an increased proliferation index in already positive cases [61]. Similarly, removal of Tregs from in vitro stimulated cell cultures, increased sensitivity of the LPA from 25 to 82% as well as the overall stimulation-index whilst preserving specificity [62]. In 15 patients with SJS/TEN, the LPA alone had a sensitivity of only 27%, whilst a combined approach evaluating granulysin expression in CD4+ T-cells, together with a granzyme B enzyme-linked immunosorbent spot (ELIspot) and IFNγ production, provided a sensitivity of 80% and specificity of 95% [63]. Similarly, Polak et al. compared LPA with cytokine assays in the acute phase in 43 patients with DHR during and/or after the acute phase and observed a high specificity: 95% for the LPA, 83% for IFNγ detection and 92% for IL-4 detection, with a sensitivity of 82% for the combined IFNγ and IL-4 detection versus only 50% for the LPA in the acute setting [64].

In order to evaluate the profile of drug-activated T-cells, alternative techniques have been developed such as flow cytometry–based methods using carboxyfluorescein diacetate succinimidyl ester staining alone [60] or in combination with 5-bromo-2-deoxyuridine [53] enabling the specific characterization of the fraction of proliferating cells in the LPA. The evaluation of more surface markers of T-cell activation (e.g. CD69, CD107a, CD40L and HLA-DR), and the evaluation of cytokine secretion using the antibody-based ELIspot assay to quantify the number of cytokine-secreting cells in antigen-stimulated PBMCs have been studied as well [1]. The latter has the advantage that low numbers, as low as 1:30,000, of drug-specific T-cells can be detected [65]. A panel of ELIspot assays to detect IFNγ, IL-4, IL-5, IL-13, IL-17, IL-22, FasL, TNF-α, granzyme B, and perforin producing T-cells is currently being investigated [1]. Most of these assays resulted in improved sensitivities whilst preserving specificity, indicating considerable progress in the in vitro diagnosis of DTH [64].

Next, Adachi et al. suggested combined testing for DTH using the basophil activation test and LPA and observed that samples that yielded positive results for LPA and basophil activation test did not overlap, suggesting that the two analyses might compensate each other for false-negativity. Combined sensitivity was higher compared with the sensitivity for the single assays (NPV of 14.7% for the basophil activation test, 28.2% for the LPA, and 96.4% for the combination) [66].

Recently, an alternative assay using the determination of IL-6 production early (20 min) after in vitro exposure of PBMCs to suspected drugs in a heterogeneous patient population (both immediate and DTH were evaluated) was reported to have a sensitivity and specificity of respectively 85 and 82%, yet it awaits further validation [67].

## Conclusions

In conclusion, considerable progress has been made in our understandings of the disease mechanisms in various DTH, with the best examples coming from HLA-associated DHR with abacavir, carbamazepine and allopurinol. Whether or not these findings can be extrapolated to other DTH remains to be elucidated. The complex interplay between host, drug and other potential factors such as infectious diseases and/or environmental factors remains to be explored. Nevertheless, pharmacogenomics studies have paved the way for pre-treatment screening for potentially severe DHR and further work in this field is highly anticipated. Also, improvements in the in vitro diagnostics will most likely enhance our understandings of DTH, and aid in the daily clinical practice of diagnosis and proper management of drug hypersensitivities.

## Abbreviations

DHR: drug hypersensitivity reactions
DTH: non-immediate/delayed-type DHR
MPE: maculopapular eruptions
DRESS: drug reaction with eosinophilia and systemic symptoms
DIHS: drug-induced hypersensitivity syndrome
AGEP: acute generalized exanthematous pustulosis
FDE: fixed drug eruptions
SJS: Stevens–Johnson syndrome
TEN: toxic epidermal necrolysis
SCARs: severe cutaneous adverse reactions
p-i: pharmacological interaction
LPA: lymphocyte proliferation assay
HLA: human leucocyte antigen
TCR: T-cell receptors
NPV: negative predictive value
PPV: positive predictive value
HHV: human herpes virus
Trm: tissue-resident memory T-cells
Tregs: regulatory T-cells
PBMCs: peripheral blood mononuclear cells
ELIspot: enzyme-linked immunosorbent spot
